# Protective action of *Bacillus clausii* probiotic strains in an in vitro model of *Rotavirus* infection

**DOI:** 10.1038/s41598-020-69533-7

**Published:** 2020-07-28

**Authors:** Lorella Paparo, Lorella Tripodi, Cristina Bruno, Laura Pisapia, Carla Damiano, Lucio Pastore, Roberto Berni Canani

**Affiliations:** 1 Department of Translational Medical Science-Pediatric Section, University of Naples “Federico II”, Via S. Pansini 5, 80131 Naples, Italy; 2 CEINGE-Biotecnologie Avanzate S.C.Ar.L., University of Naples Federico II, Naples, Italy; 3 Department of Molecular Medicine and Biotechnology, University of Naples Federico II, Naples, Italy; 4 European Laboratory for the Investigation of Food-Induced Diseases, University of Naples Federico II, Naples, Italy; 5 Task Force on Microbiome Studies, University of Naples Federico II, Naples, Italy

**Keywords:** Gastroenteritis, Viral infection

## Abstract

*Rotavirus* is the most common cause of acute gastroenteritis (AGE) in young children. *Bacillus clausii* (*B. clausii*) is a spore-forming probiotic that is able to colonize the gut. A mixture of four *B. clausii* strains (O/C, T, SIN and N/R) is commonly used for the treatment of AGE, and it has been demonstrated that it can reduce the duration and severity of diarrhea in children with AGE. Few studies have sought to characterize the mechanisms responsible for such beneficial effects. Intestinal effects of probiotics are likely to be strain-specific. We conducted a series of in vitro experiments investigating the activities of this mixture of *B. clausii* strains on biomarkers of mucosal barrier integrity and immune function in a cellular model of *Rotavirus* infection. *B. clausii* protected enterocytes against *Rotavirus*-induced decrease in trans-epithelial electrical resistance, and up-regulated expression of mucin 5AC and tight junction proteins (occludin and zonula occludens-1), all of which are important for effective mucosal barrier function. *B. clausii* also inhibited reactive oxygen species production and release of pro-inflammatory cytokines (interleukin-8 and interferon-β) in *Rotavirus*-infected cells, and down-regulated pro-inflammatory Toll-like receptor 3 pathway gene expression. Such mechanisms likely contributed to the observed protective effects of *B. clausii* against reduced cell proliferation and increased apoptosis in *Rotavirus*-infected enterocytes.

## Introduction

Acute gastroenteritis (AGE), defined as sudden-onset diarrhea that is unrelated to chronic disease, with or without nausea, vomiting, fever or abdominal pain, is disproportionately common among young children^1,2^. *Rotavirus* (RV) is the most common cause of AGE and the leading cause of AGE-associated mortality in children younger than 5 years of age^2–6^. In 2016, more than 258 million episodes of diarrhea and approximately 1.5 million hospitalizations and 128,500 deaths in children younger than 5 years were attributable to RV infection globally^2,7^. The highest rates of RV-associated mortality have been reported in sub-Saharan Africa, Southeast Asia, and South Asia^7^. The high cost of RV vaccination precludes its widespread use in such low-income settings^8^. However, even in developed countries, AGE remains a considerable burden, despite the implementation of RV vaccination programs^7^. For example, routine RV vaccination was introduced in 2006 in the US, but there were 70,553 AGE-associated hospital admissions, about 20,000 due to RV infection, among US children younger than 5 years in 2013, which were associated with direct costs of more than US $226 million^9^.

Probiotics are living microorganisms that, when administered in adequate amounts, confer a health benefit on the host after colonizing the gut, and can help to prevent and treat AGE by supporting a healthy gut and immune system^10,11^. Short- and long-term beneficial effects of probiotics on the gut are the result of a range of mechanisms, including competitive exclusion and direct antagonism of gut pathogens, stimulation of host mucosal immune mechanisms, and reconstitution and enhancement of intestinal barrier function^3,11,12^. However, not all such beneficial effects can be ascribed to probiotics as a general class, as effects occurring at the intestinal or extra-intestinal level are likely to be strain-specific^11^.

*Bacillus clausii* (*B. clausii*) is a rod-shaped, spore-forming, aerobic, Gram-positive probiotic bacterium that is acid resistant and able to colonize the gut^13–16^. Data suggest that a mixture of four *B. clausii* probiotic strains (O/C, T, SIN and N/R) is effective in the treatment of pediatric AGE^13^. General antimicrobial and immunomodulatory properties of these *B. clausii* strains have been previously described^17^, but specific mechanisms of action against AGE are still largely undefined.

The current study aimed to investigate the protective activities of a mixture of four *B. clausii* strains (O/C, T, SIN and N/R) and their metabolites, on human enterocytes in basal conditions and in a model of RV infection. The effects of *B. clausii* on indicators of mucosal barrier integrity and innate immune function were also examined.

## Results

### Human beta defensin 2 and cathelicidin synthesis

**Figure 1:**
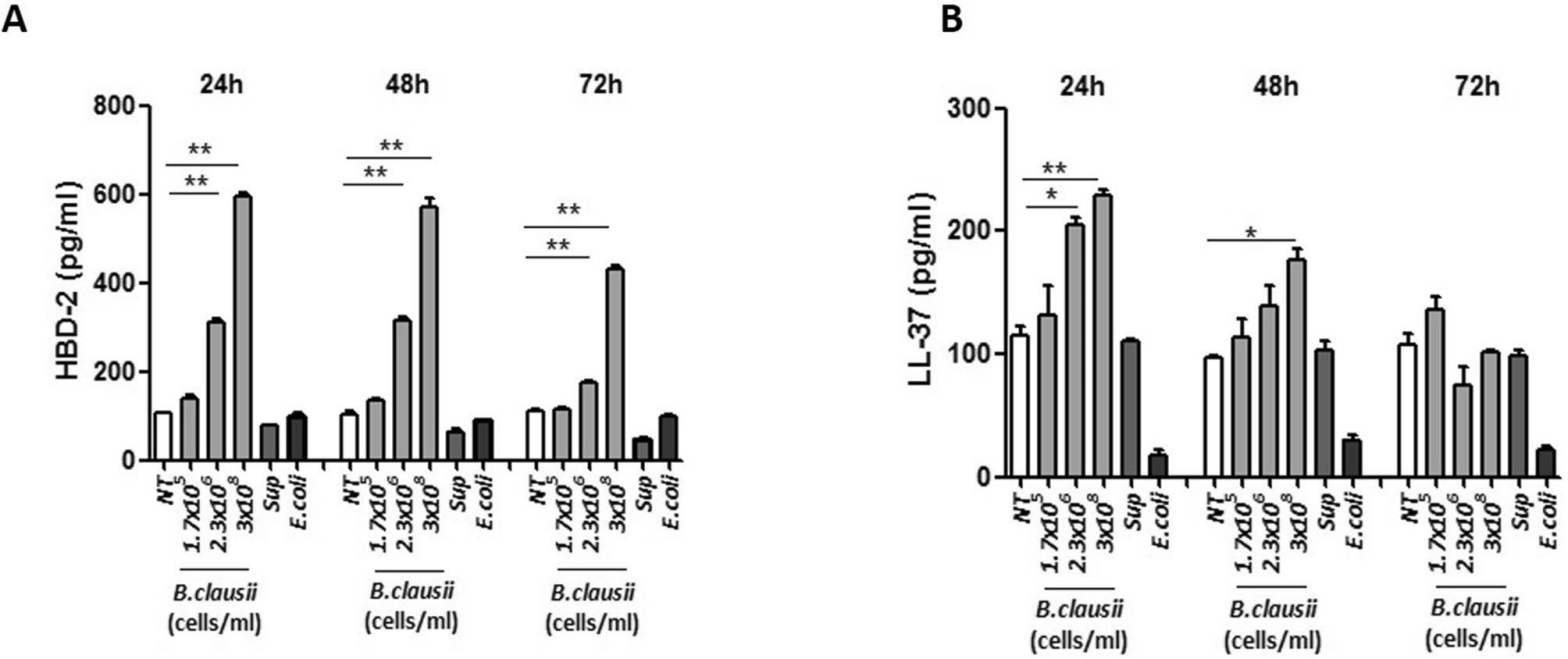
*B. clausii* increases HBD-2 and LL-37 expression in human enterocytes. Cells were exposed to *B. clausii* probiotic strains mix at different concentrations; *B. clausii* supernatant (Sup, dilution 1:100) or *E. coli* K12 (1 × 10^6^ cells/mL) as control. Only the exposure to *B. clausii* strains was able to elicit a significant increase in HBD-2 (**A**) and LL-37 (**B**) production by human enterocytes. HBD 2, human beta defensin 2; LL-37, cathelicidin; NT, untreated. *p < 0.05 vs NT; **p < 0.001 vs NT.

Relative to untreated enterocytes, *B. clausii* strains, but not its supernatant, elicited a dose-dependent increase of HBD-2 and LL-37 synthesis (Fig. 1A,B). Maximal effects were obtained after 48 h of treatment with *B. clausii* strains 3 × 10^8^ cells/mL (*P* < 0.001 vs untreated cells).

### Proliferation, cell cycle and apoptosis analysis by flow cytometry

**Figure 2:**
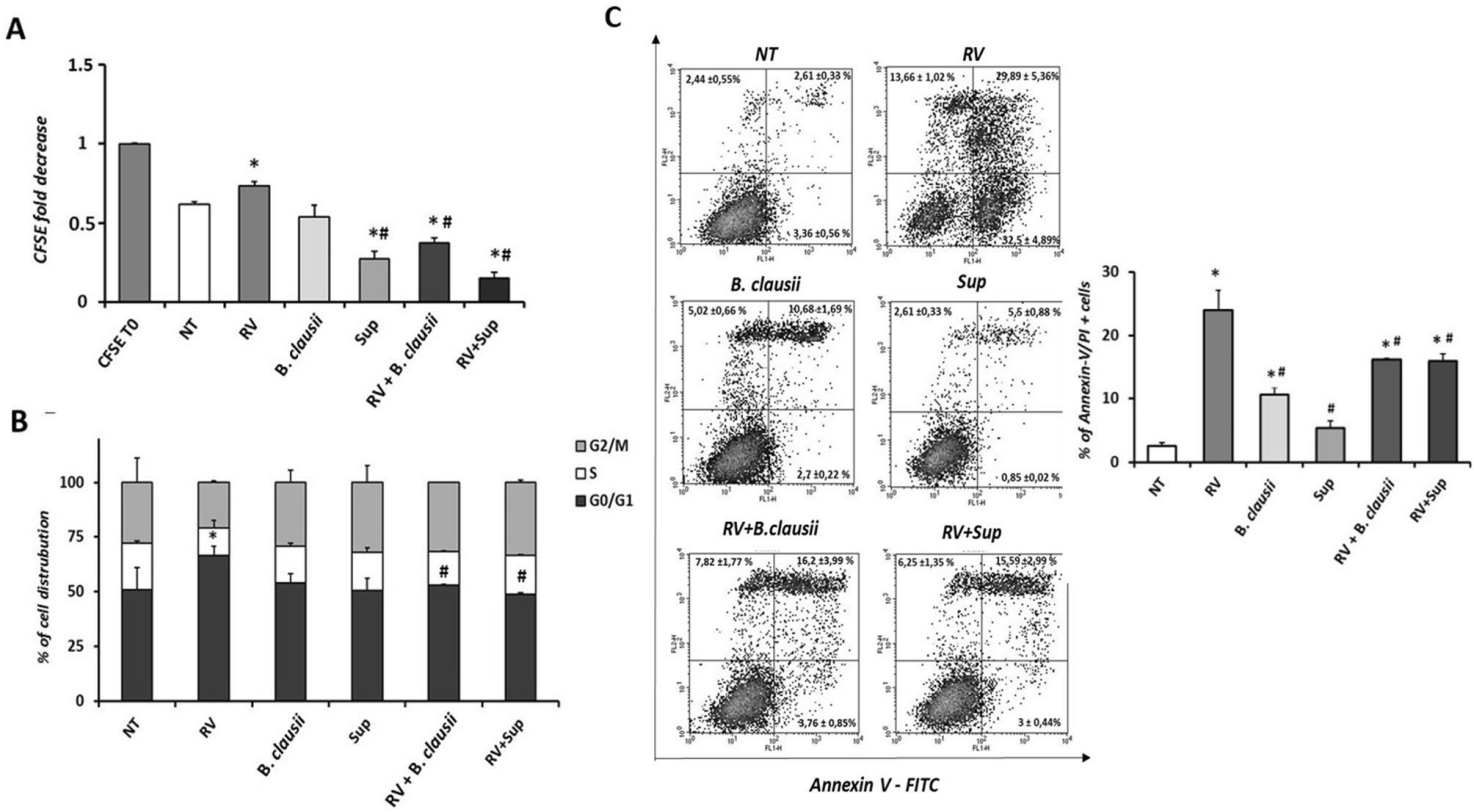
*B. clausii* counteracts the Rotavirus effects on human enterocytes proliferation and viability. (**A**) Rotavirus (RV) (10 pfu/cell) reduced human enterocytes proliferation rate. *B. clausii* probiotic strains (3 × 10^8^ cells/mL) and *B. clausii* supernatant (Sup, dilution 1:100) were able to inhibit the RV effect. (**B**) Cell cycle analysis confirmed the reduction in proliferation and a block in G0/G1 phases induced by RV. Again, the effect was inhibited by the incubation with *B. clausii* probiotic strains (3 × 10^8^ cells/mL) and *B. clausii* supernatant (dilution 1:100). (**C**) Apoptosis analysis showed that the exposure to RV resulted in pro-apototic effect on human enterocytes. Again, both *B. clausii* and its supernatant were able to inhibit this effect. *p < 0.05 vs NT, ^#^p < 0.05 vs RV.

After 24 h of treatment with *B. clausii* probiotic strains or *B. clausii* supernatant, cell proliferation was comparable to that of the untreated cells, whereas RV significantly reduced cell growth (Fig. 2A). The combination of RV with *B. clausii* probiotic strains or *B. clausii* supernatant partially restored the proliferation rate (*P* < 0.05 vs RV alone). Rotavirus exposure blocked proliferation, with almost 70% of cells arrested in G0/G1 phase (Fig. 2B). We observed a G1/S transition block with RV compared with untreated cells and compared with infected cells stimulated with *B. clausii* probiotic strains or *B. clausii* supernatant for 24 h (*P* < 0.05). Compared with RV alone, greater proportions of cells exposed to a combination of RV and *B. clausii* probiotic strains or *B. clausii* supernatant were in the G2/M phase. Double staining with Annexin V and PI to evaluate apoptosis induction showed a toxic effect of RV stimulation (Fig. 2C), as demonstrated by an increase in necrotic cells (positive only for PI) and late apoptotic cells (positive for both PI and Annexin V) relative to untreated cells and uninfected cells treated with *B. clausii* probiotic strains or *B. clausii* supernatant. Treatment of RV*-*infected cells with *B. clausii* strains or *B. clausii* supernatant reduced the proportion of necrotic and apoptotic cells.

### Transepithelial electrical resistance

**Figure 3:**
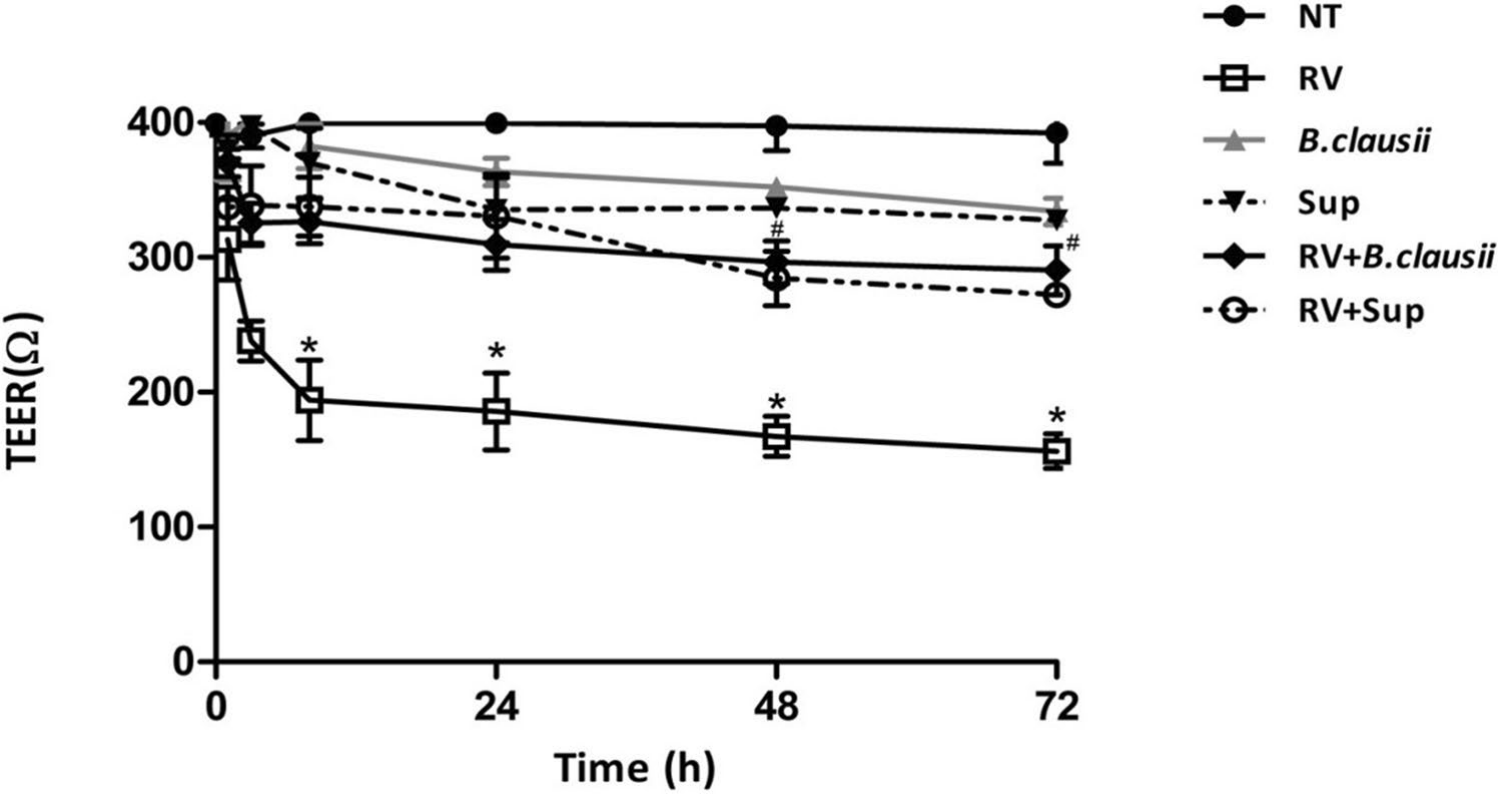
*B. clausii* and its supernatant significantly inhibit Rotavirus-induced TEER reduction in human enterocytes. The incubation with Rotavirus (RV) (10 pfu/cell), but not with *B. clausii* probiotic strains (3 × 10^8^ cells/mL) or with *B. clausii* supernatant (Sup, dilution 1:100), elicited a significant reduction of TEER. *B. clausii* probiotic strains (3 × 10^8^ cells/mL) and *B. clausii* supernatant (Sup, dilution 1:100) significantly inhibited the RV-induced TEER decrease. *p < 0.05 vs NT; ^#^p < 0.05 vs RV.

Treatment of uninfected cells with *B. clausii* probiotic strains or with *B. clausii* supernatant did not affect TEER, but RV-infected cells had decreased TEER (*P* < 0.05 vs untreated cells from 8 to 72 h; Fig. 3). Stimulation with *B. clausii* probiotic strains or *B. clausii* supernatant protected against a RV-induced decrease in TEER (*P* < 0.05 vs RV alone from 8 to 72 h).

### ROS production

**Figure 4:**
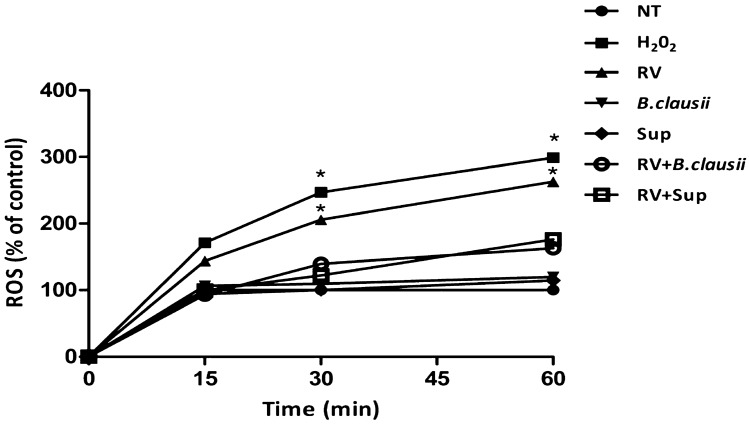
*B. clausii* and its supernatant significantly inhibit Rotavirus-induced ROS production in human enterocytes. Rotavirus (RV) (10 pfu/cell), but not with *B. clausii* probiotic strains (3 × 10^8^ cells/mL) or with *B. clausii* supernatant (Sup, dilution 1:100), induced a significant increase in ROS production in a time-dependent manner. *B. clausii* probiotic strains (3 × 10^8^ cells/mL) and *B. clausii* supernatant (Sup, dilution 1:100) significantly inhibited the RV-induced increase in ROS. H_2_O_2_ as a positive control. *p < 0.05 vs NT.

Rotavirus significantly increased ROS production in a time-dependent manner (Fig. 4). *B. clausii* probiotic strains and *B. clausii* supernatant inhibited the RV-induced increase in ROS.

### Expression of MUC5AC and tight junction proteins

**Figure 5:**
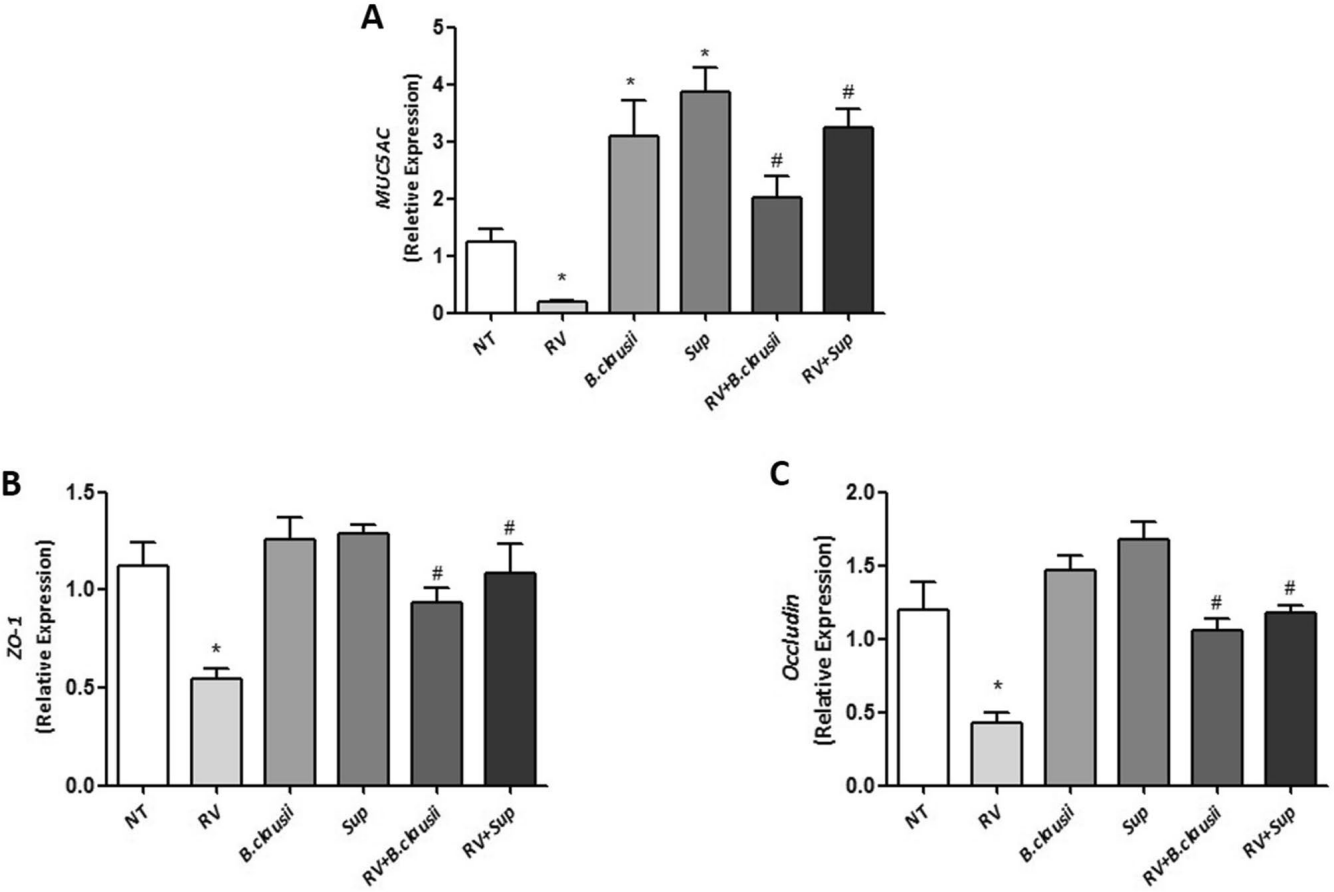
*B. clausii* and its supernatant significantly counteract the Rotavirus-induced alteration in MUC-5, ZO-1 and Occludin expression in human enterocytes. Rotavirus (RV) (10 pfu/cell) significantly reduced the expression of MUC5AC (**A**), ZO-1 (**B**) and Occludin (**C**) in human enterocytes, whereas both *B. clausii* probiotic strains (3 × 10^8^ cells/mL) and *B. clausii* supernatant (Sup, dilution 1:100) significantly increased the expression of these proteins in basal condition and blunted the RV effect. *p < 0.05 vs NT; ^#^p < 0.05 vs RV.

Compared with untreated cells, *B. clausii* probiotic strains and *B. clausii* supernatant significantly increased MUC5AC expression under basal conditions, while RV infection alone significantly reduced MUC5AC expression (Fig. 5A). *B. clausii* probiotic strains and *B. clausii* supernatant upregulated MUC5AC expression in RV-infected cells (*P* < 0.05 vs RV alone).

Rotavirus infection significantly reduced ZO-1 and occludin expression (Fig. 5B,C). The combination of RV with *B. clausii* probiotic strains or *B. clausii* supernatant significantly increased expression of both tight junction proteins expression levels. These results were confirmed when analyzed the protein for occludin and ZO-1, as showed in Fig. S1A,B.

### Analysis of interleukin-8 and interferon-β production

**Figure 6:**
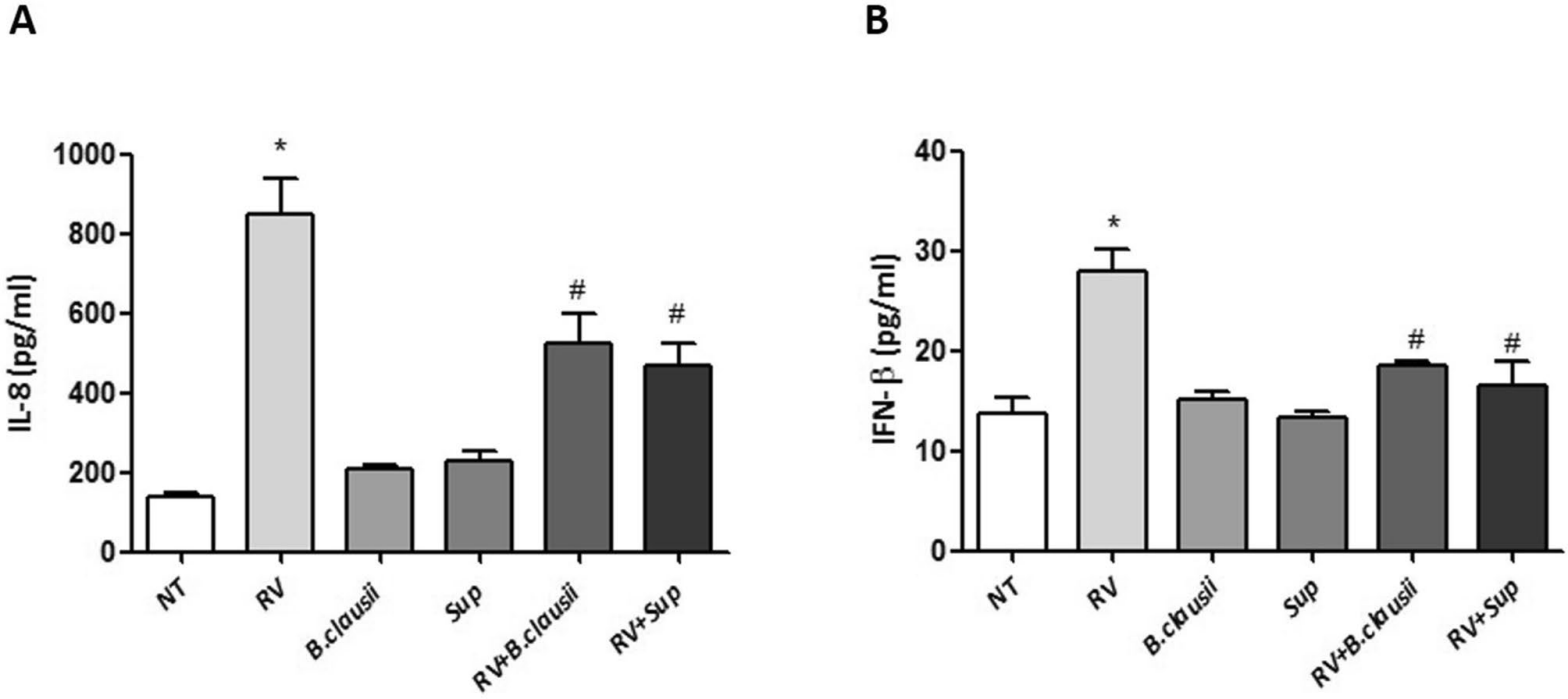
*B. clausii* and its supernatant significantly reduce Rotavirus-induced expression of pro-inflammatory cytokines in human enterocytes. Rotavirus (RV) (10 pfu/cell) elicited a significant increase in IL-8 (**A**) and IFN-β (**B**) production. *B. clausii* probiotic strains (3 × 10^8^ cells/mL) and *B. clausii* supernatant (Sup, dilution 1:100) significantly inhibited the RV-induced increase in IL-8 and IFN-β production. *p < 0.05 vs NT; ^#^p < 0.05 vs RV.

There was a significant increase in IL-8 and IFN-β production in RV-infected enterocytes (Fig. 6A,B). These effects were blunted by *B. clausii* probiotic strains and *B. clausii* supernatant (*P* < 0.05 vs RV alone).

### Toll-like receptor 3 pathway analysis

**Figure 7:**
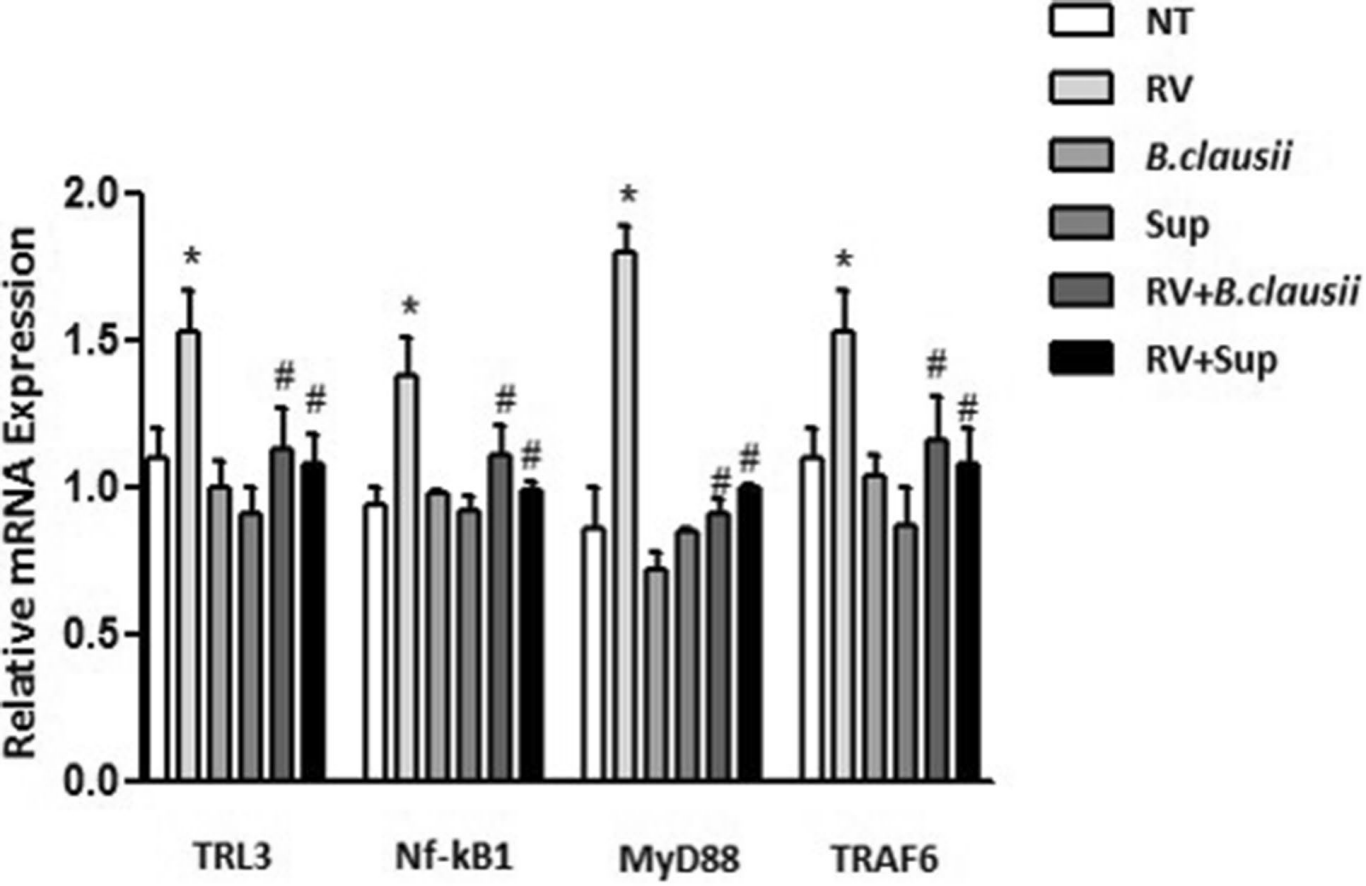
*B. clausii* and its supernatant contrast Rotavirus-mediated activation of Toll-like receptor-3 pathway in human enterocytes. Rotavirus (RV) (10 pfu/cell) significantly up-regulated TLR3, NF-κB1, MyD88 and TRAF6 expression. *B. clausii* probiotic strains (3 × 10^8^ cells/mL) and *B. clausii* supernatant (Sup, dilution 1:100) significantly inhibited such effects. *p < 0.05 vs NT; ^#^p < 0.05 vs RV.

TLR3, NF-κB1, MyD88 and TRAF6 expression were all significantly up-regulated in cells infected with RV compared with untreated cells (Fig. 7). Down-regulation of pro-inflammatory TLR3 pathway gene expression was observed in RV-infected cells treated with *B. clausii* probiotic strains and *B. clausii* supernatant (*P* < 0.05 vs RV alone). The respective proteins of these genes showed the same trend of mRNA expression (Fig. S2A,B).

## Discussion

Children are constantly being exposed to infectious agents in the gastrointestinal tract and are able to resist these infections due to the action of two main defense mechanisms: the non-immune mucosal barrier and immune response^18,19^. The results of our study provide evidence that a mixture of four *B. clausii* probiotic strains, namely O/C, T, SIN, and N/R, and their metabolites modulate a number of non-immune and immune defense mechanisms against RV-induced AGE.

To determine whether treatment with *B. clausii* probiotic strains and their supernatant induce a protective action against RV infection, we evaluated enterocyte proliferation and survival. Rotavirus reduced cell growth in association with a cytotoxic effect. We demonstrated that *B. clausii* probiotic strains and their metabolites (from the supernatant) were able to restore cell proliferation, inducing a restart in cell cycle progression and protecting against apoptosis, in RV-infected human enterocytes.

The non-immune mucosal barrier acts through a well-modulated network involving epithelial cell layers that express tight cell–cell contacts (tight-junctions regulated by several proteins including occludin and ZO-1), and the secreted mucus layer that overlays the epithelium in the intestinal tract^20^. The integrity of the non-immune mucosal barrier, which is of pivotal importance in protecting children against pathogens, is disrupted by RV^21^. We found that *B. clausii* probiotic strains and their metabolites had beneficial effects on markers of epithelial barrier damage and enterocyte monolayer permeability in cells infected by RV. Whereas *B. clausii* probiotic strains or supernatant did not affect epithelial integrity as assessed by TEER in uninfected cells, they protected infected cells against a RV-induced increase in TEER. *B. clausii* probiotic strains and supernatant also up-regulated the expression of mucin protein MUC5AC and occluding and ZO-1 tight junction proteins, all of which are important for effective mucosal barrier function^20^.

The intestinal epithelium is an integral component of innate immunity, which provides the initial host response to invading pathogens^22^. Soluble proteins and bioactive small molecules are involved in the innate immune response. These are either constitutively present at a systemic level in many biological fluids (e.g., complement proteins and defensins) or are released from activated cells resulting in inflammation (e.g., cytokines, reactive free radical species and bioactive amines)^22^. In experiments conducted in Caco-2 enterocytes under basal conditions, *B. clausii* probiotic strains and supernatant increased the synthesis of the innate immunity antimicrobial peptides HBD-2 and LL-37, which are responsible for effective defense mechanisms against several pathogens in the gastrointestinal tract^23^. *B. clausii* probiotic strains and supernatant also inhibited ROS production, and the release of pro-inflammatory cytokines (IL-8 and IFN-β) in RV-infected cells.

The innate immune system also includes membrane-bound receptors and cytoplasmic proteins that bind invading microbes^22^. For example, RV double stranded RNA binds TLR3, expressed in intestinal epithelial cells, and this interaction elicits an upregulation of the expression of type I IFN (IFN-α, IFN-β)^24–26^ that are crucial to limit RV infection^27^ to block cell replication, and a secretion of various cytokines and chemokines, including IL-8 and IFN-β^22^. We showed that *B. clausii* probiotic strains were able to reduced secretion of IL-8 and IFN-β, and down-regulate the expression of pro-inflammatory TLR3 pathway genes (TLR3, NF-κB1, MyD88, TRAF6) activate by RV infection. Our results are consistent with clinical trials^13^, and help to explain the beneficial effects of the same mixture of four *B. clausii* probiotic strains (O/C, T, SIN, N/R) in children with AGE. In a meta-analysis of randomized controlled trials, the same *B. clausii* strains mixture reduced the duration of diarrhea by 9.12 h relative to control (oral rehydration solutions or placebo), and duration of hospitalization was reduced by 0.85 days^13^. There was also a trend towards a reduction in stool frequency. In line with the findings of the meta-analysis, data from a large observational study indicated a reduction in diarrhea duration (median duration 3 days) and stool frequency in children with viral or antibiotic-associated AGE who were treated with the O/C, T, SIN, N/R mix of *B. clausii* strains^28^. There was no significant difference in the mean duration of diarrhea between patients with viral diarrhea and those with antibiotic-associated diarrhea. The *B. clausii* O/C strain has previously been reported to produce bacteriocin and protease antimicrobials with activity against Gram-positive bacteria, which may be partially responsible for the protective effects of this probiotic in antibiotic-associated diarrhea^17,29^ .

Although a specific recommendation for *B. clausii* in the treatment of childhood AGE has hitherto been missing because of limited data, there is now a growing body of clinical evidence in support of the O/C, T, SIN, N/R mix of *B. clausii* probiotic strains as an effective therapeutic option in this clinical setting^13,28,30^. Modulation of the immune response with this *B. clausii* strains mix may also have health benefits beyond the treatment of AGE, ranging from the prevention of recurrent respiratory infections in allergy-prone children to influencing outcomes in cancer patients^31,32^. In addition to its documented efficacy and safety in childhood AGE^13,28^, *B. clausii* has the practical advantage of being a spore-forming probiotic, making it heat stable and able to be transported and stored at room temperature without loss of viability^33^.

Given that certain effects of probiotics, including immunomodulatory effects, are likely to be strain-specific^11^, a key strength of our study and the aforementioned clinical studies is that a clearly defined mix of four *B. clausii* strains was used. Just as the clinical effects of a particular strain of probiotic should not be extrapolated to other strains, intestinal mechanisms of action should not be ascribed to all strains^8,11^. Another strength of our study is the use of a validated in vitro model of enterocyte infection with RV^34^, which is the most relevant AGE-causing pathogen in the pediatric age worldwide^2,7^. Additionally, we assessed a wide range of variables that are indicative of intestinal mucosal barrier integrity and innate immune function. As well as testing a mixture of four *B. clausii* strains, we tested the effects of its supernatant on all variables. This is important to assess as it is often mainly the metabolites produced by probiotics could modulate intestinal epithelial cell functions^35^.

The main limitation of our study is related to the fact that we did not explore the potential protective effects of *B. clausii* against other viral and non-viral agents responsible for AGE in the pediatric age group. Evaluation of the efficacy and mechanisms of action of *B. clausii* probiotic strains on more complex systems, such as human biopsies and/or organoids exposed to different gastrointestinal pathogens, is advocated to further explore the potential of such therapeutic approach.

## Conclusion

The mixture of four *B. clausii* probiotic strains investigated in this study has protective effects and stimulates various non-immune mucosal barrier and innate immune system defense mechanisms in a human enterocyte model of RV infection. These observations provide insights into the mechanisms that are potentially responsible for the beneficial effects of this *B. clausii* mixture in pediatric patients with AGE, and should encourage further study into the effects of this probiotic on AGE caused by other viral and non-viral pathogens.

## Methods

### *B. clausii* probiotic strains and supernatant

The commercially available mixture of *B. clausii* strains investigated in this study (Enterogermina) had the following composition: O/C (3 × 10^8^), T (1.7 × 10^5^), SIN (2.3 × 10^6^), N/R (1.7 × 10^7^). *B. clausii* supernatant was prepared as previously described^32^. Briefly, *B. clausii* supernatant was obtained by centrifugation and filtration of a suspension of the four *B. clausii* probiotic strains (3.2 × 10^8^ cells/mL), which was cultured aerobically in a specific fermentation medium at 37 °C in a rotary shaker for 3 days. *B. clausii* supernatant was harvested by centrifugation at 4 °C (10,000*g* for 5 min) and filtered onto 0.2 μm cellulose membrane.

### Cell line

All experiments were conducted using the Caco-2 cell line of human enterocytes (American Type Culture Collection, Middlesex, UK; accession number: HTB-37). Cells were grown to confluence in Dulbecco’s modified Eagle’s medium (Gibco, Berlin, Germany) supplemented with 20% fetal bovine serum (FBS; Lonza, Visp, Switzerland), 1% l-glutamine (Lonza), 1% non-essential amino acids, and 1% penicillin/streptomycin (Lonza). Cells were cultured at 37 °C in a water-saturated atmosphere consisting of 95% air and 5% CO_2_. The medium was changed every 2 days and Caco-2 cells were grown for 14 days after confluence and cultured in 6-well plates.

### Rotavirus strain and infection protocol

The simian RV strain SA11 was used as previously described^30^. Briefly, the virus (10 pfu/cell) was activated with 20 µg/mL porcine trypsin for 1 h at 37 °C. The viral suspension was added to the apical side of Caco-2 cell monolayers. After 1 h, the cells were washed and incubated in FBS-free medium for the indicated time periods after infection.

### *B. clausii* cell stimulation protocol

For dose–response and time-course experiments, uninfected Caco-2 cells were stimulated for 24, 48 and 72 h with the mixture of four *B. clausii* probiotic strains (O/C, T, SIN and N/R) at three different doses (1.7 × 10^5^, 2.3 × 10^6^, 3 × 10^8^ cells/mL), serial dilutions of *B. clausii* supernatants or medium only (untreated cells). Serial dilutions of *B. clausii* supernatants were prepared in phosphate buffered saline (PBS) as previously described^36^. *E. coli* K12 strain (1 × 10^6^ cells/mL) served as control.

For RV infection experiments, Caco-2 cells were pre-treated for 12 h with *B. clausii* probiotic strains (3 × 10^8^ cells/mL) or *B. clausii* supernatant (dilution 1:100). The cells were then washed in PBS and activated RV was added in FBS-free medium for 1 h at 37 °C. Infected cells were then re-suspended in medium with *B. clausii* probiotic strains (3 × 10^8^ cells/mL) or its supernatant (dilution 1:100) for 24 or 48 h. Infected and uninfected cells that were not pretreated with *B. clausii* probiotic strains or *B. clausii* supernatant were incubated in medium alone.

### Human beta defensin 2 and cathelicidin synthesis

Concentrations of the antimicrobial peptides human beta defensin 2 (HBD-2) and cathelicidin LL-37 in the cell supernatant were determined using commercially available enzyme-linked immunosorbent assay (ELISA) kits specific for human HBD-2 (Phoenix Pharmaceuticals, Inc., Burlingame, CA, USA) or LL-37 (Hycult biotechnology, Uden, The Netherlands), respectively. ELISA was performed according to the manufacturers’ recommendations.

### Proliferation, cell cycle and apoptosis analysis by flow cytometry

To perform proliferation analysis, undifferentiated Caco-2 cells were stained with carboxyfluorescein succinimidyl ester (CFSE) at a final concentration of 2.5 μM (Cell-Trace CFSE Proliferation Kit, Molecular Probes, Invitrogen, Carlsbad, CA, USA) according to the manufacturer’s protocol. Briefly, cells were incubated with CFSE 2.5 μM for 15 min in darkness, stirring occasionally. The reaction was stopped by adding PBS with 20% FBS followed by centrifugation and repeated twice. Cells were then counted and 0.5 × 10^6^ cells/well were plated in 6-well plates, stimulated and analyzed after 24 h.

To perform cell cycle analysis, 0.5 × 10^6^ un-differentiated Caco-2 cells were plated in 6-well plates. After stimulation for 24 h, cells were collected and stained with propidium iodide (PI) 50 μg/mL (Sigma-Aldrich, St. Louis, MO, USA) in the presence of RNase A 100 μg/mL (Serva, Heidelberg, Germany).

To analyze cell apoptosis rates, Annexin V Apoptosis Detection Kit APC was used (eBioscience; San Diego, CA, USA) according to the manufacturer’s protocol. After 48 h of treatment, the cells were washed with PBS and incubated with 1 × Annexin V binding buffer, then 5 × 10^5^ cells were stained with Annexin V-fluorescein isothiocyanate (FITC) for 10 min at room temperature in the dark. Before reading with a BD FACS Calibur flow cytometer flow cytometer (Becton Dickinson, Franklin Lakes, NJ, USA), PI 5 μg/mL was added.

### Transepithelial electrical resistance measurement

The Caco-2 cell monolayer transepithelial electrical resistance (TEER) was measured using a Millicel-ERS resistance monitoring apparatus (Merck Millipore, Billerica, MA, USA). TEER was measured at 1, 3, 8, 24, 48, and 72 h after Rotavirus infection.

### Reactive oxygen species production

Reactive oxygen species (ROS) production was measured using 7′-dichlorofluorescein diacetate (DCFH-DA) spectrofluorometry. After stimulation, cells were exposed to DCFH-DA 20 µL (D6665; Sigma-Aldrich, St. Louis, MO, USA) for 30 min at 37 °C in the dark, with 10 mM of H_2_O_2_ (Sigma-Aldrich) used as a positive control. Intracellular ROS production was measured using a fluorometer (SFM 25; Kontron Instruments; Japan).

### Quantitative real-time polymerase chain reaction

Quantitative real-time polymerase chain reaction (qRT‐PCR) was used to analyze the effect of intestinal exposure to *B. clausii* probiotic strains on gene expression of mucin 5AC (MUC5AC) and the tight junction proteins occludin and zonula occludens-1 (ZO-1), as well as toll-like receptor 3 (TLR3), nuclear factor κ B subunit 1 (NF-κB1), myeloid differentiation primary response 88 (MyD88), and tumor necrosis factor receptor-associated factor 6 (TRAF6), as previously described^37^. Briefly, qRT‐PCR was performed with the TaqMan gene expression assay kit, (Applied Biosystems; Grand Island, NY, USA) according to the manufacturer's instructions. Samples were run in duplicate at 95 °C for 15 s and 60 °C for 1 min using an ABI Prism 7900 Sequence Detection System (Applied Biosystems). Data were analyzed using the comparative threshold cycle method. We used the glyceraldehyde 3-phosphate dehydrogenase (GAPDH) gene to normalize the level of mRNA expression.

### Western blot analysis

Total proteins were extracted from Caco-2 cells using Trizol reagent (Invitrogen, Life Technologies, CA, USA) following the manufacturer’s instructions. The concentrations were determined by using a protein assay kit adopting bovine serum albumin standards, according to the manufacturer’s instructions (Bio-Rad Laboratories, Hercules, CA, USA). A total of 30 μg protein was separated by SDS-polyacrylamide gel electrophoresis and blots were prepared on a nitrocellulose membrane Amersham Hybond-ECL (Amersham, GEhealthcare, USA). The membranes was then incubated with anti-occludin (#31721, AbCam, Cambridge, UK), anti-ZO-1 (#96587, AbCam), anti-TRL3 (PA520183, Thermofisher, Waltham, MA, USA) anti-TRAF6 (#40675, AbCam), anti-MyD88 (#133739, AbCam), anti-NF-κB1 (#13586, Cell signaling Technology, Danvers, MA, USA) and anti-β actin (ACTBD11B7, Santa Cruz, CA, USA) primary antibodies at different dilutions. A goat anti-rabbit or anti-mouse horseradish peroxidase-linked antibody (Amersham, Orsay, France) was used at a 1:2,000 and 1:500 dilution, respectively, as a secondary antibody. Bound immunoglobulins was revealed by the ECL-detection system and the quantification was performed by Chemidoc (Biorad, Hercules, CA, USA).

### Analysis of interleukin-8 and interferon-β production

Interleukin 8 (IL-8) production was measured in the cell supernatant using a commercially available ELISA kit specific for IL-8 (Abcam; Cambridge, UK). Interferon-β (IFN-β) concentration was measured using the specific human ELISA assay kit (BioVendor; Brno, Czech Republic). All ELISA assays were performed according to the manufacturer’s recommendations, and results expressed as pg/mL.

### Statistical analysis

All experiments were performed in triplicate and were repeated twice. The Kolmogorov–Smirnov test was used to determine whether variables were normally distributed. We used the *t* test to evaluate differences among continuous variables. The level of significance for all statistical tests was 2-sided, P < 0.05. All analyses were conducted in SPSS version 16.0 for Windows (SPSS Inc., Chicago, IL, USA) and GraphPad Prism 5 (La Jolla, CA, USA)^37^.

## Supplementary information

Supplementary Figures.

## Data Availability

The datasets generated during and/or analyzed during the current study are available from the corresponding author on reasonable request.

## References

[1] Hartman S, Brown E, Loomis E, Russell HA (2019). Gastroenteritis in children. Am. Fam. Physician.

[2] G. B. D. Diarrhoeal Disease Collaborators (2018). Estimates of the global, regional, and national morbidity, mortality, and aetiologies of diarrhoea in 195 countries: a systematic analysis for the Global Burden of Disease Study 2016. Lancet Infect. Dis..

[3] Cameron D (2017). Probiotics for gastrointestinal disorders: Proposed recommendations for children of the Asia-Pacific region. World J. Gastroenterol..

[4] Chiejina, M. & Samant, H. Viral Diarrhea. In *StatPearls* (2020). 29262044

[5] Lanata CF (2013). Global causes of diarrheal disease mortality in children <5 years of age: a systematic review. PLoS ONE.

[6] Nirwati H (2019). Norovirus and rotavirus infections in children less than five years of age hospitalized with acute gastroenteritis in Indonesia. Arch. Virol..

[7] Troeger C (2018). Rotavirus vaccination and the global burden of rotavirus diarrhea among children younger than 5 years. JAMA Pediatr..

[8] Szajewska H (2014). Use of probiotics for management of acute gastroenteritis: a position paper by the ESPGHAN Working Group for Probiotics and Prebiotics. J. Pediatr. Gastroenterol. Nutr..

[9] Leshem E (2018). National estimates of reductions in acute gastroenteritis-related hospitalizations and associated costs in us children after implementation of rotavirus vaccines. J. Pediatr. Infect. Dis. Soc..

[10] Berni Canani R (2007). Probiotics for treatment of acute diarrhoea in children: randomised clinical trial of five different preparations. BMJ.

[11] Hill C (2014). Expert consensus document. The International Scientific Association for Probiotics and Prebiotics consensus statement on the scope and appropriate use of the term probiotic. Nat. Rev. Gastroenterol. Hepatol..

[12] Guarnier F (2012). World Gastroenterology Organisation Global Guidelines: probiotics and prebiotics October 2011. J. Clin. Gastroenterol..

[13] Ianiro G (2018). *Bacillus**clausii* for the treatment of acute diarrhea in children: a systematic review and meta-analysis of randomized controlled trials. Nutrients.

[14] Cutting SM (2011). *Bacillus* probiotics. Food Microbiol..

[15] Elshaghabee FMF, Rokana N, Gulhane RD, Sharma C, Panwar H (2017). *Bacillus* as potential probiotics: status, concerns, and future perspectives. Front. Microbiol..

[16] Ghelardi E (2015). Survival and persistence of *Bacillus**clausii* in the human gastrointestinal tract following oral administration as spore-based probiotic formulation. J. Appl. Microbiol..

[17] Urdaci MC, Bressollier P, Pinchuk I (2004). *Bacillus**clausii* probiotic strains: antimicrobial and immunomodulatory activities. J. Clin. Gastroenterol..

[18] Allaire JM (2018). The intestinal epithelium: Central coordinator of mucosal immunity. Trends Immunol..

[19] Takiishi T, Fenero CIM, Camara NOS (2017). Intestinal barrier and gut microbiota: shaping our immune responses throughout life. Tissue Barriers.

[20] Capaldo CT, Powell DN, Kalman D (2017). Layered defense: how mucus and tight junctions seal the intestinal barrier. J. Mol. Med. (Berl)..

[21] Guttman JA, Finlay BB (2009). Tight junctions as targets of infectious agents. Biochim. Biophys. Acta.

[22] Chaplin DD (2010). Overview of the immune response. J. Allergy Clin. Immunol..

[23] Cobo ER, Chadee K (2013). Antimicrobial human beta-defensins in the colon and their role in infectious and non-infectious diseases. Pathogens.

[24] Sen A (2012). Signal transducer and activator of transcription 3 (STAT3) and survivin induction by varicella-zoster virus promote replication and skin pathogenesis. Proc. Natl. Acad. Sci. U.S.A.

[25] Meylan E, Tschopp J (2006). Toll-like receptors and RNA helicases: two parallel ways to trigger antiviral responses. Mol. Cell.

[26] Kawai T, Akira S (2006). Innate immune recognition of viral infection. Nat. Immunol..

[27] Lin JD (2016). Distinct roles of type I and type III interferons in intestinal immunity to homologous and heterologous rotavirus infections. PLoS Pathog..

[28] de Castro JA, Guno MJV, Perez MO (2019). *Bacillus**clausii* as adjunctive treatment for acute community-acquired diarrhea among Filipino children: a large-scale, multicenter, open-label study (CODDLE). Trop. Dis. Travel Med. Vaccines.

[29] Ripert G (2016). Secreted compounds of the probiotic *Bacillus clausii* strain O/C inhibit the cytotoxic effects induced by clostridium difficile and *Bacillus**cereus* toxins. Antimicrob. Agents Chemother..

[30] Guarino A (2014). European Society for Pediatric Gastroenterology, Hepatology, and Nutrition/European Society for Pediatric Infectious Diseases evidence-based guidelines for the management of acute gastroenteritis in children in Europe: update 2014. J. Pediatr. Gastroenterol. Nutr..

[31] Marseglia GL (2007). Efficacy of *Bacillus clausii* spores in the prevention of recurrent respiratory infections in children: a pilot study. Ther. Clin. Risk Manag..

[32] Riquelme E (2019). Tumor microbiome diversity and composition influence pancreatic cancer outcomes. Cell.

[33] Jayanthi N, Ratna Sudha M (2015). *Bacillus clausii*—the probiotic of choice in the treatment of diarrhoea. J. Yoga Phys. Ther..

[34] De Marco G (2009). Rotavirus induces a biphasic enterotoxic and cytotoxic response in human-derived intestinal enterocytes, which is inhibited by human immunoglobulins. J. Infect. Dis..

[35] De Marco S (2018). Probiotic cell-free supernatants exhibited anti-inflammatory and antioxidant activity on human gut epithelial cells and macrophages stimulated with lPS. Evid. Based Complement Alternat. Med..

[36] Medrano M, Perez PF, Abraham AG (2008). Kefiran antagonizes cytopathic effects of *Bacillus**cereus* extracellular factors. Int. J. Food Microbiol..

[37] Paparo L (2018). Direct effects of fermented cow's milk product with *Lactobacillus**paracasei* CBA L74 on human enterocytes. Benef. Microbes.

