# Supplementary material for: Protective action of Bacillus clausii probiotic strains in an in vitro model of Rotavirus infection
Source: Sci Rep. 2020 Jul 28;10:12636. doi: 10.1038/s41598-020-69533-7 (PMC7387476; doi:10.1038/s41598-020-69533-7)
Supplement: Supplementary file 1 — Supplementary Figures. [file 41598_2020_69533_MOESM1_ESM.docx]

**Supplementary Figures**

**Title: Protective action of *Bacillus clausii* probiotic strains in an *in vitro* model of *Rotavirus* infection**

**Authors**: Lorella Paparo, Lorella Tripodi, Cristina Bruno, Laura Pisapia, Carla Damiano, Lucio Pastore, and Roberto Berni Canani

**
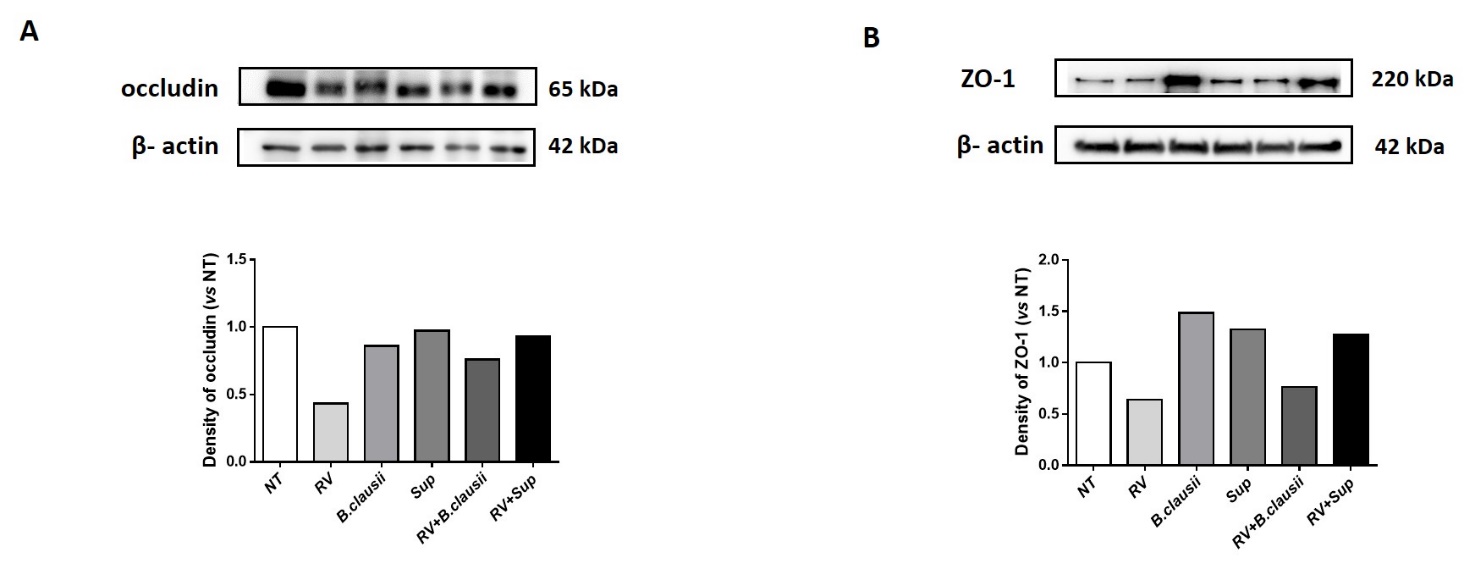
**

**Figure S1. Occludin and ZO-1 protein analysis.** Representative image of three experiments qualitatively similar. Western blot assay of occludin **(A)** and ZO-1 **(B)** was performed on protein extracts from Caco-2 cells. The amounts of occludin, ZO-1 and β-actin were measured by Western blot. The histogram below shows optical density of the proteins, obtained with Image Lab software. Relative quantification of proteins was normalized versus β-actin protein and was calculated using NT as calibrator. *B. clausii, Bacillus clausii* (3 x 10^8^ cells/mL); NT, untreated; RV, *Rotavirus* (10 pfu/cell); Sup, supernatant (dilution 1:100); ZO-1, zonula occludens -1.


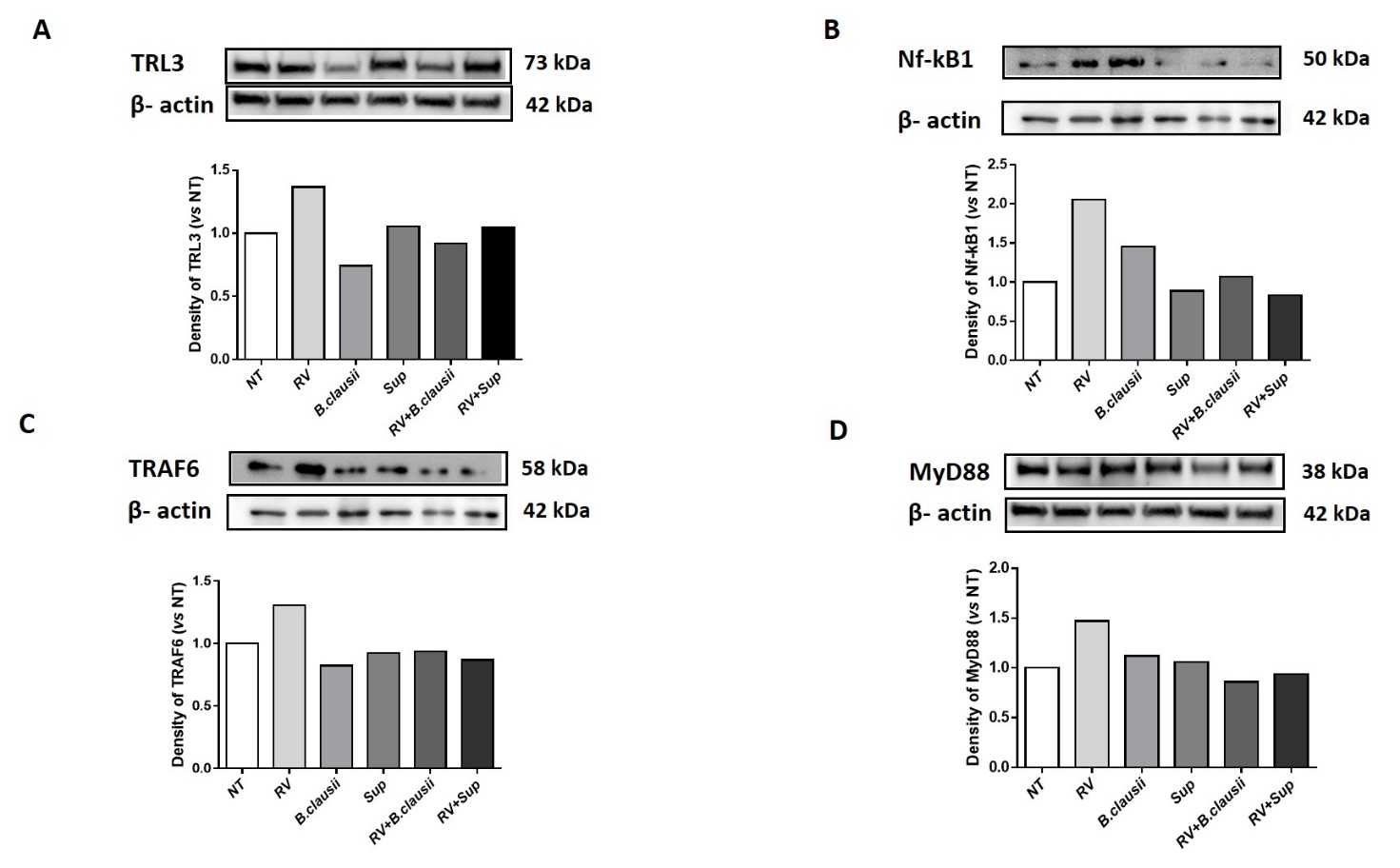


**Figure S2. TLR3, NF-κB1, MyD88 and TRAF6 protein analysis.** Representative image of three experiments qualitatively similar. Western blot assay of TLR3 **(A)**, NF-κB1 **(B)**, MyD88 **(C)** and TRAF6 **(D)** was performed on protein extracts from Caco-2 cells. The amounts of these proteins and of β-actin were measured by Western blot. The histogram below shows optical density of the proteins obtained with Image Lab software. Relative quantification of proteins was normalized versus β-actin protein and was calculated using NT as calibrator. *B. clausii, Bacillus clausii* (3 x 10^8^ cells/mL); MyD88, myeloid differentiation primary response 88; NF-κB1, nuclear factor κ B subunit 1; NT, untreated; RV, *Rotavirus* (10 pfu/cell); Sup, supernatant (dilution 1:100); TRL3, toll-like receptor 3 (TRL3); TRAF6, tumor necrosis factor receptor-associated factor 6.

**Supplementary Information**

**Full-length gel of Occludin, β-actin and TRAF6**

Occludin and β-actin **(A)**, and TRAF6 **(B)**was blotted on the same gel (PW: 65, 42 and 58 kDa, respectively). At left side of the image was represented the molecular weight markers (#161-0373 Precision Plus Protein Standards Biorad, Hercules, CA, USA). TRAF6 **(B)** was detected after western blot membrane stripping protocol (<https://www.abcam.com/protocols/western-blot-membrane-stripping-for-restaining-protocol>).

Starting from the left side of the gel:

**Lane 1:** NT, untreated

**Lane 2:** RV, *Rotavirus* (10 pfu/cell)

**Lane 3:** *B. clausii, Bacillus clausii* (3 x 10^8^ cells/mL)

**Lane 4:** Sup, supernatant (dilution 1:100)

**Lane 5:** *B. clausii, Bacillus clausii* (3 x 10^8^ cells/mL) + RV

**Lane 6**: Sup, supernatant (dilution 1:100) + RV

******Last lane represented a testing sample to set up the procedure*

**(A)**

**
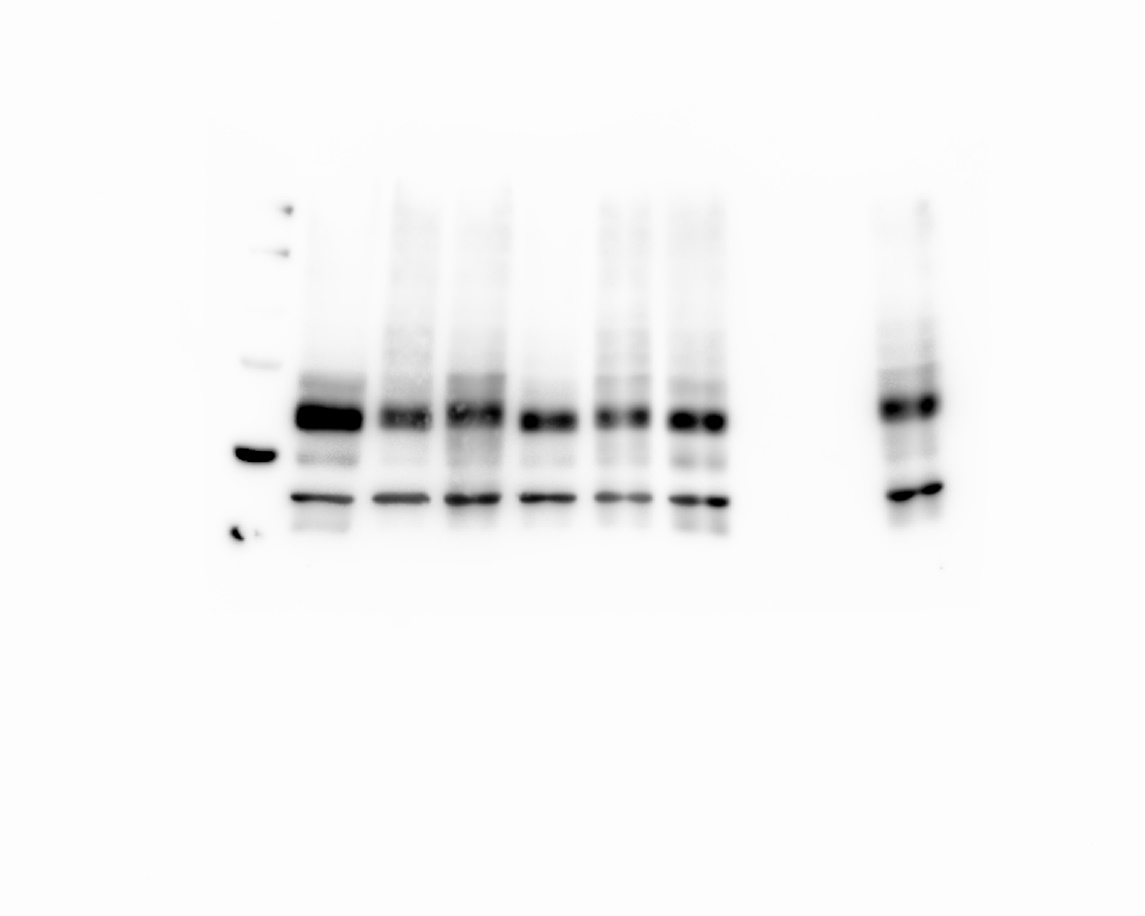
**

37kDa

50kDa

75kDa

**(B)**

**
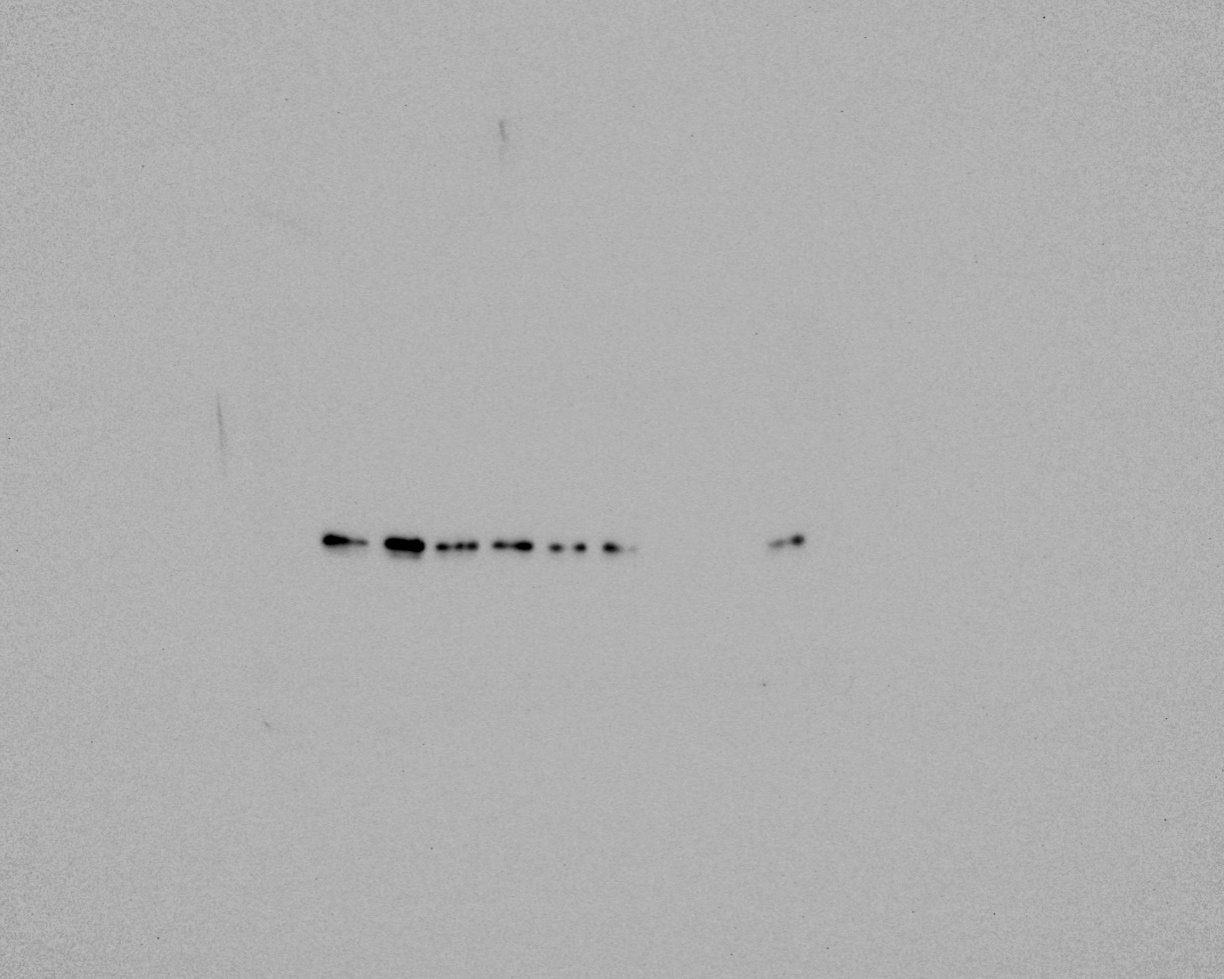
**

75kDa

50kDa

**Full-length gels of Zonula occludens-1 (ZO-1) and β-actin**

ZO-1**(A)** and β-actin **(B)** was blotted on the same gel and the cropped at the specific size of each protein (240 and 42 kDa, respectively). At left side of the image was represented the rainbow molecular weight markers, full-range Mr 12000 to 225000, ten separate proteins with six different colors (#RPN800E, GE Healthcare, Amersham Pl, UK).

Starting from the left side of the gel:

**Lane 1:** NT, untreated

**Lane 2:** RV, *Rotavirus* (10 pfu/cell)

**Lane 3:** *B. clausii, Bacillus clausii* (3 x 10^8^ cells/mL)

**Lane 4:** Sup, supernatant (dilution 1:100)

**Lane 5:** *B. clausii, Bacillus clausii* (3 x 10^8^ cells/mL) + RV

**Lane 6**: Sup, supernatant (dilution 1:100) + RV

**(A)**

**
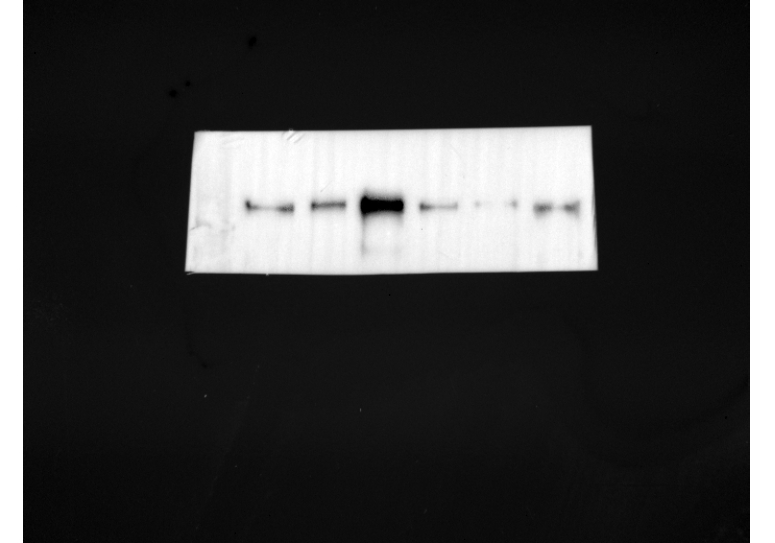
**

225kDa

**(B)**

**
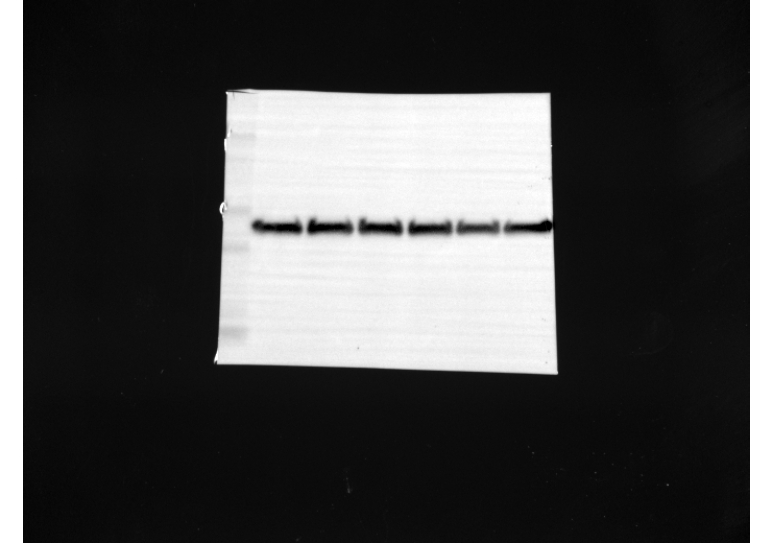
**

38KDa

24KDa

**Full-length gels of Nf-kB1 and β-actin**

Nf-kB1 **(A)**and β-actin **(B)** was blotted on the same gel and the cropped at the specific size of each protein (50 and 42 kDa, respectively). At left side of the image was represented the molecular weight markers (#161-0373 Precision Plus Protein Standards Biorad, Hercules, CA, USA).

Starting from the left side of the gel:

**Lane 1:** NT, untreated

**Lane 2:** RV, *Rotavirus* (10 pfu/cell)

**Lane 3:** *B. clausii, Bacillus clausii* (3 x 10^8^ cells/mL)

**Lane 4:** Sup, supernatant (dilution 1:100)

**Lane 5:** *B. clausii, Bacillus clausii* (3 x 10^8^ cells/mL) + RV

**Lane 6**: Sup, supernatant (dilution 1:100) + RV

**(A)**


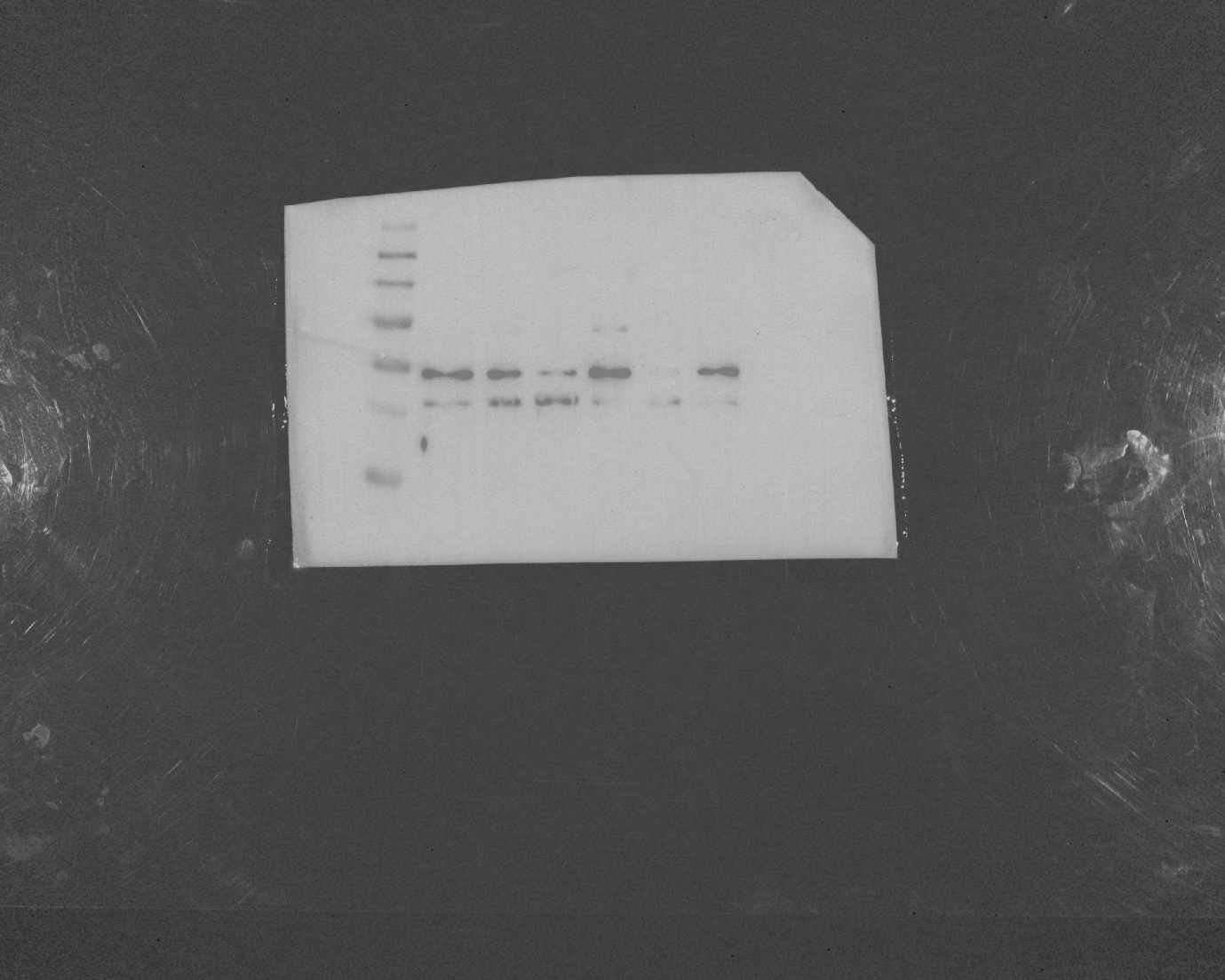


50kDa

**(B)**


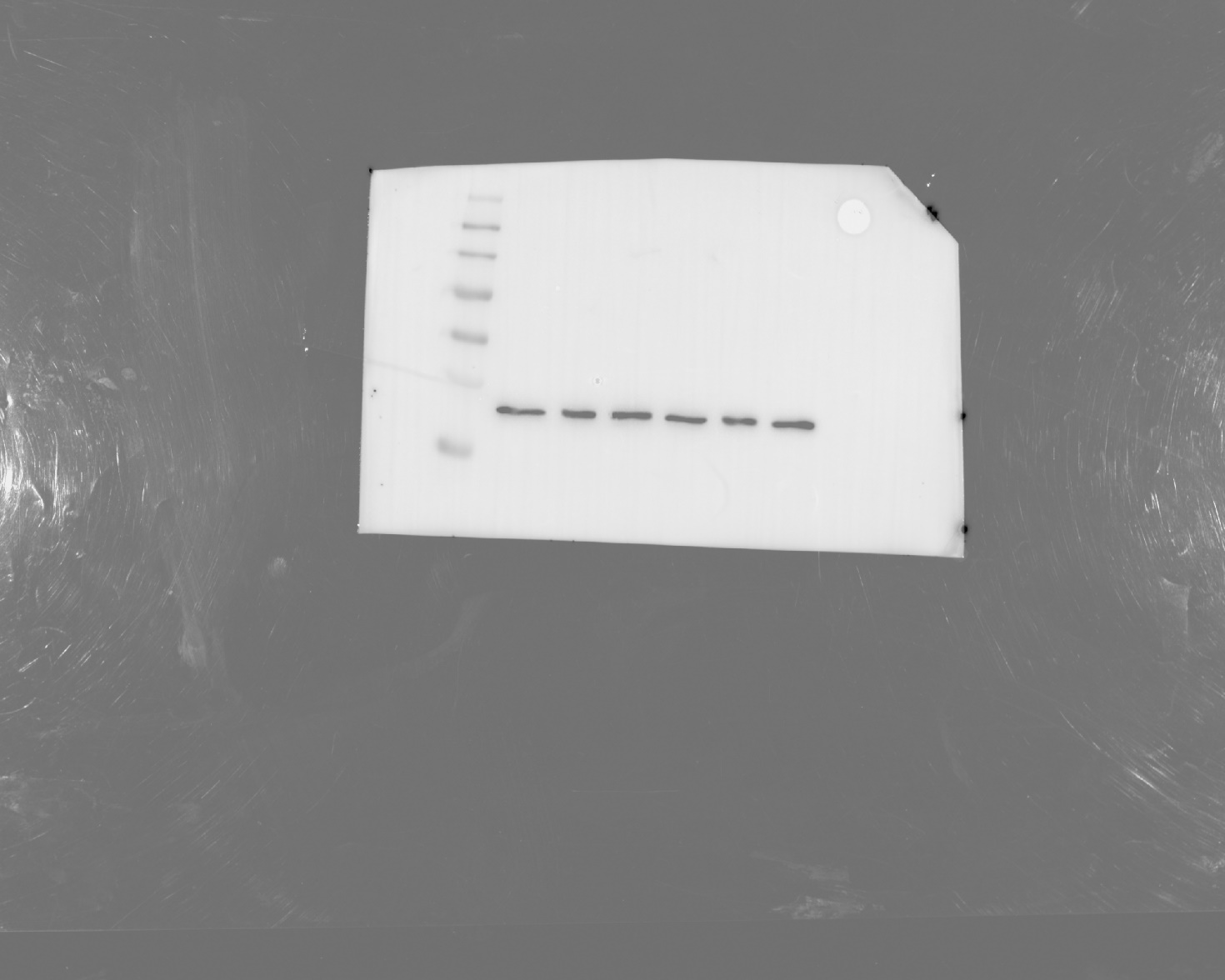


50kDa

37kDa

**Full-length gels of TRL3, MyD88 and β-actin**

TRL3 **(A)**, MyD88 **(B)** and β-actin **(C)** was blotted on the same gel and the cropped at the specific size of each protein (73, 38 and 42 kDa, respectively). At left side of the image was represented the rainbow molecular weight markers, full-range Mr 12000 to 225000, ten separate proteins with six different colors (#RPN800E, GE Healthcare, Amersham Pl, UK).

Starting from the left side of the gel:

**Lane 1:** NT, untreated

**Lane 2:** RV, *Rotavirus* (10 pfu/cell)

**Lane 3:** *B. clausii, Bacillus clausii* (3 x 10^8^ cells/mL)

**Lane 4:** Sup, supernatant (dilution 1:100)

**Lane 5:** *B. clausii, Bacillus clausii* (3 x 10^8^ cells/mL) + RV

**Lane 6**: Sup, supernatant (dilution 1:100) + RV

**(A)**


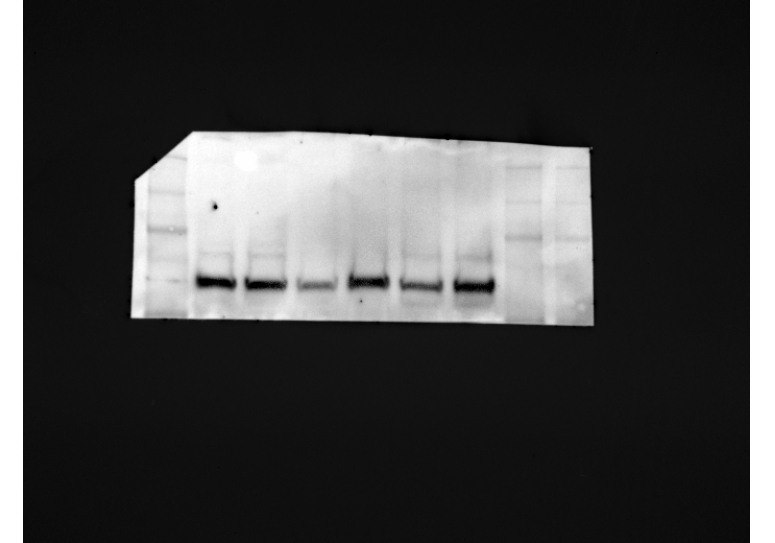


72KDa

**(B)**


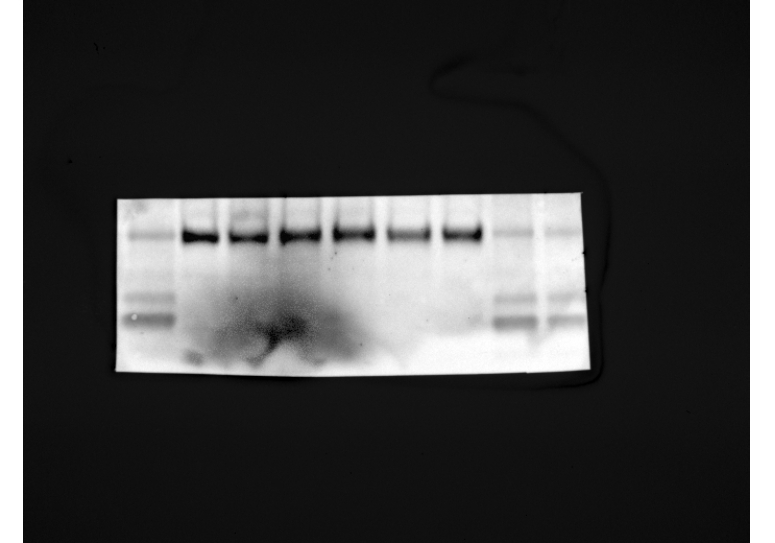


24KDa

38KDa

**(C)**


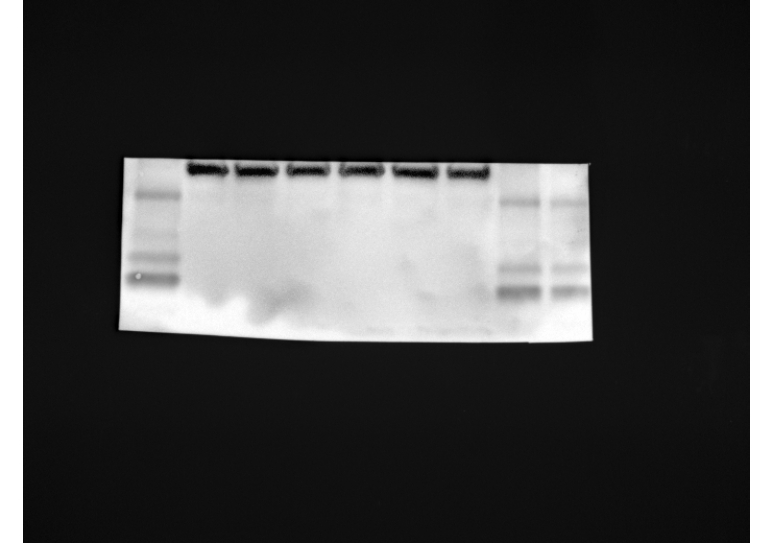


24KDa

38KDa
